# Subcutaneous Injections of Air or Water Neuralgia

**Published:** 1915-08

**Authors:** 


**Subcutaneous Injections of Air or Water in Neuralgia.** Grasset and Rimbaud, Journ. de Méd. et de Chir. prat., lxxxiv, 15. The object is to free the nerve-endings held fast in the hyperaemic tissues. Surmont and Dubois make use of distilled water, Debove and Bruhl use saline (75 per cent.) or Hayem's serum: sodium chloride 5, sodium sulphate 10, sterilized water 100. The injections are given at the painful spots in a dose of from 5 to 10 c.c. repeated every two or three days. Sciatica can be cured by ten to fifteen injections. Cordier, of Lyons, was the first to use injections of air for neuralgia, using an apparatus similar to Paquelin's cautery, in which the cautery point is replaced by a needle, the air being filtered through a tube plugged with absorbent wool. The pump of Potain's aspirator may be used in like manner. The seats of election for the injections in the case of sciatica are the upper part of the buttock, the middle and after parts of the thigh, and the outer side of the leg. After having put in the needle, it is necessary to make sure that the point is not in a vessel. Air is then pumped in very gently, and the patient should only feel some numbness and tingling. The air spreads out irregularly under the skin, makes the limb torpid, and reaches the loins and the popliteal space. From 500 to 2,000 c.c. are thus injected, and the gas is absorbed in from two days to a fortnight. The method is extremely simple, harmless and painless; the patient is nearly always relieved at once. Intercostal neuralgia may be treated in the same may; femoro-cutaneous neuralgia has been relieved; while good results have been obtained in the diffuse painful neuritis, following severe injuries like contusions of the shoulder and the hip. In obstinate cases of sciatica, Sicard advises a combination of injections of air, and of water, and epidural injections. He injects from 800 to 1,200 c.c. of air at the level of the leg; from 60 to 80 c.c. of normal saline containing about ½ cg. of novocain immediately below the sciatic notch in the upper part of the ischio-trochanteric groove; and finally, from 10 to 20 c.c. of the same solution into the lower sacro-lumbar epidural region.

